# Health Risk or Resource? Gradual and Independent Association between Self-Rated Health and Mortality Persists Over 30 Years

**DOI:** 10.1371/journal.pone.0030795

**Published:** 2012-02-09

**Authors:** Matthias Bopp, Julia Braun, Felix Gutzwiller, David Faeh, for the Swiss National Cohort Study Group

**Affiliations:** Institute of Social and Preventive Medicine (ISPM), University of Zurich, Zurich, Switzerland; Innsbruck Medical University, Austria

## Abstract

**Background:**

Poor self-rated health (SRH) is associated with increased mortality. However, most studies only adjust for few health risk factors and/or do not analyse whether this association is consistent also for intermediate categories of SRH and for follow-up periods exceeding 5–10 years. This study examined whether the SRH-mortality association remained significant 30 years after assessment when adjusting for a wide range of known clinical, behavioural and socio-demographic risk factors.

**Methods:**

We followed-up 8,251 men and women aged ≥16 years who participated 1977–79 in a community based health study and were anonymously linked with the Swiss National Cohort (SNC) until the end of 2008. Covariates were measured at baseline and included education, marital status, smoking, medical history, medication, blood glucose and pressure.

**Results:**

92.8% of the original study participants could be linked to a census, mortality or emigration record of the SNC. Loss to follow-up 1980–2000 was 5.8%. Even after 30 years of follow-up and after adjustment for all covariates, the association between SRH and all-cause mortality remained strong and estimates almost linearly increased from “excellent” (reference: hazard ratio, HR 1) to “good” (men: HR 1.07 95% confidence interval 0.92–1.24, women: 1.22, 1.01–1.46) to “fair” (1.41, 1.18–1.68; 1.39, 1.14–1.70) to “poor”(1.61, 1.15–2.25; 1.49, 1.07–2.06) to “very poor” (2.85, 1.25–6.51; 1.30, 0.18–9.35). Persons answering the SRH question with “don't know” (1.87, 1.21–2.88; 1.26, 0.87–1.83) had also an increased mortality risk; this was pronounced in men and in the first years of follow-up.

**Conclusions:**

SRH is a strong and “dose-dependent” predictor of mortality. The association was largely independent from covariates and remained significant after decades. This suggests that SRH provides relevant and sustained health information beyond classical risk factors or medical history and reflects salutogenetic rather than pathogenetic pathways.

## Introduction

“How in general would you rate your health?”. For decades this question has been asked in health surveys. Soon, good self-rated health (SRH) was found to be associated with better survival [1]. Already in the early 1980s SRH proved to be a strong predictor of mortality, even independently of objective health parameters [1]. Since then, associations between SRH and objective health endpoints have been confirmed by dozens of population studies and in different cultural environments [1]–[4]. Although the general association between SRH and death appears to be universal, its strength may depend on the cultural understanding of health and differ between countries and men and women, but also by age, socio-economic position (SEP) and the time passed between assessment and outcome [1], [2], [5]. Medical history, functional status, health behaviour and subjective emotions appear to influence the relationship between SRH and death [1], [2]. However, due to their design, only few studies were able to consider all relevant mediators and to examine the impact of varying follow-up time on the SRH-mortality association [2]. Moreover, most studies were restricted to elderly or to dichotomized SRH, precluding examination of a “dose dependent” nature of the SRH-mortality association [2], [4], [6]–[9]. Due to generally rather short follow-up periods, previous studies were not able to convincingly rebut the suspicion that the SRH-mortality-association could arise from an individual's presentiment of conditions which were not (yet) detected by his/her physician [10].

We aimed at specifying the association between SRH and mortality in men and women and at determining to what extent this relationship was mediated by covariates. Our study is based on an anonymous record linkage that combined baseline data from a health examination conducted in five towns of Switzerland in 1977–79 with individual data from national censuses, mortality and emigration records up to the end of 2008. This resulted in a considerably large study population that could be followed up for more than 30 years. A special feature of our study is therefore the potential to analyze the impact of the assessment of lag time of outcome on the SRH-mortality association. With the large number of deaths accumulated over the observation period, we had enough power to also examine whether there is a “dose-dependence” in the relationship between the five SRH rates and mortality.

## Methods

### National Research Program 1A (NRP 1A) data

The NRP 1A was a community health promotion initiative focused on cardiovascular disease prevention [11]. It has been conducted 1977–1979 in five towns (Table 1) of Switzerland. A random sample of individuals aged 16–69 years was drawn as described [11]. Participation rate was 65.1% (Swiss nationals) and 42.2% (foreign nationals) [12]. 2,085 men and 2,301 women attended a health examination and completed a self-administered questionnaire (sampled). Additional 4,245 individuals aged ≥16 years participated spontaneously. Most of the spontaneous participants were volunteers sensitized by the public health promotion initiatives of the program and/or were family members of the sampled participants. Requirements for a mortality follow-up were missed. Encouraged by the success of a recently conducted anonymous record linkage of participants data from another study [13], we adopted the procedure to NRP 1A, using date of birth, sex, marital status and geographical information from census, mortality and migration registries. All data used for this study was anonymous (no names, addresses or PINs). Approval (Nr. 13/06) was obtained from the Ethics Committee of the Canton of Zurich (Kantonale Ethik-Kommission, KEK). The use of written informed consent was not custom at the time, the NFP 1A was conducted and the original questionnaires containing names have been deleted many years ago.

**Table 1:** NRP 1A participants (by study participation status) vs. general Swiss population, individuals aged 16–69 years.

	NRP 1A Participants 1977–79*		General population 1980**	
	Sampled	Spontaneous	Towns	Switzerland
Total population	4378	3873	62119	4332052
Town				
Aarau (N)	923	2104	11371	
Solothurn (N)	1247	–	11160	
Nyon (N)	826	1769	8932	
Vevey (N)	924	–	11242	
Lugano (N)	458	–	19414	
Age (mean)	37.8	44.2	44	42.6
Household size (mean)	2.6	2.7	2.7	3.0
Women (%)	52.1	55.0	54.0	50.7
Educational level				
Lower (%)	37.8	33.2	41.6	43.7
Intermediate (%)	44.2	49.7	40.4	41.1
Upper (%)	18.1	17.1	18.1	15.2
Marital status				
Single (%)	27.9	21.8	26.8	24.9
Married (%)	64.5	69.8	61.7	66.1
Widowed (%)	2.6	4.5	5.8	4.6
Divorced or separated (%)	5.0	3.9	5.8	4.4
Foreign nationality (%)	37.1	9.3	25.0	15.5

*N = 8,251; 92 participants with invalid date of birth and 288 participants exceeding the official upper limit of age excluded.

**figures for the general population are from the 1980 census (Swiss Federal Statistical Office).

The initial NRP 1A database encompasses 8,631 participants. Because of incomplete date of birth (N = 3, all Nyon) or an obvious misentry “January, 1” and no additional household member among the participants (N = 89, all Aarau), 92 participants had to be excluded, leaving 3,928 men and 4,611 women for record linkage. Of these, 7 sampled and 281 spontaneous participants exceeded the upper age limit set in the original study protocol (69 years), but were nevertheless included in the analysis.

### Swiss National Cohort (SNC) data

The SNC encompasses all residents of Switzerland enumerated in the national 1990 or 2000 census. Deterministic and probabilistic record linkage methods were used to link anonymized census records to death or emigration records; details were described [13], [14]. As exact date of birth was not available for the 1980 census, standard linkage of 1980 census records is often not possible due to insufficient specifity of the remaining identification variables. This especially applies to younger persons and singles. Swiss census enumeration and registration of deaths occurring in Switzerland (including cause of death information) pass for being virtually complete (presumably >99%) [14]. Registration of deaths – but not necessarily of cause of death – of Swiss nationals abroad should be fairly complete. However, for foreign nationals residing in Switzerland, registration of deaths occurring abroad is incomplete.

### Linkage of NRP 1A and SNC

In order to determine vital status of NRP 1A participants, we used record linkage procedures including all potential identification variables, i.e. variables available as well in NRP 1A and in the census. Minimal requirement was sex, exact date of birth and study town. Additionally helpful were nationality, marital status, educational category and religious affiliation. A special feature of NRP 1A supported successful linkage even when specificity of single individual information was low: 4,359 (51%) of all NRP 1A participants available for linkage had at least one additional family member in the study population. We therefore started linkage by trying to match families as a whole and utilizing census information concerning other family members.

Information about community of residence five years before the census helped to retrieve NRP 1A participants who moved between study entry and census day. Deaths which occurred before the 1990 census were not covered by the SNC, and had to be evaluated separately for potential linkage. Therefore, for deaths occurred before the 1990 census, linkage success may be slightly lower. A few study participants could not be traced in the 1990 census (i.e. in the baseline SNC). However, additional linkage efforts allowed to link many of them to the 1980 or the 2000 census or a death record, thus enabling a follow-up qualifying them as successful links.

Record linkage between NRP 1A and SNC was performed step by step, with satisfactorily linked individuals excluded from succeeding steps:

1. *NRP 1A town = current or 1985 community of residence according to the 1990 census*
2. *Families only: surrounding of NRP 1A town = current or 1985 community of residence according to the 1990 census*
3. *NRP 1A town or surrounding = community of residence according to mortality statistics*
4. *Singles only: surrounding of NRP 1A town = current or 1985 community of residence according to the 1990 census*
5. *check of remaining unlinked NRP 1A participants for potential partner records in the 1980 and 1990 census and the 1977–90 death records*
6. *NRP 1A town = current or 1995 community of residence according to the 2000 census*
7. *1990 and 2000 census records linked to NRP 1A participants in steps 1) to 6): transfer of SNC mortality follow-up information 1990–2008*

**Figure 1:**
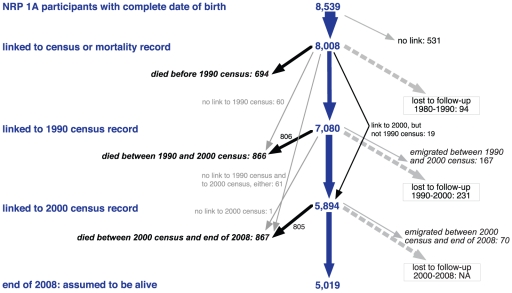
NRP 1A 1977–79 and Swiss National Cohort (SNC) until 2008: chart of linked participants. NRP: National Research Program. NA: not available, can only be determined when 2010 census will be linked.

Overall, 8,008 out of 8,539 eligible (93.8%) or 8,631 original (92.8%) NRP 1A participants could be followed-up for mortality between 40 days and almost 32 years (mean: 25.8 y), accumulating 206,644 person-years and 2,427 deaths (Figure 1). For 7,080 of these a link to the 1990 census was found and for 5,894 a link to the 2000 census (5,875 to both, 1990 and 2000 censuses). For 94 participants only a link to the 1980 census, but not to a subsequent record (1990/2000 census, death or emigration) could be established. Overall 2,427 individuals could be linked to a death record and 237 to an emigration record. Including 167 matches with emigration records, loss to follow-up between 1980 and 2000 amounted to 5.8%. Since there was no census at the end of the study, loss to follow-up after the 2000 census could not be determined, i.e. all 5,019 individuals linked to the 2000 census but not to a succeeding death or emigration record were assumed to have survived.

### Covariates

The following question was used to assess SRH: “How would you describe your state of health in general?”; possible answers were (in this order): “excellent”; “quite good”; “fair”; “rather poor”; “very poor”; “I don't know”. For adjustment, we selected the following variables from the large number of available variables from the NRP 1A: sex, town of residence, age at study entry, being a sampled or spontaneous participant, nationality, educational level (lower, intermediate, upper), marital status (single, married, divorced or separated, widowed), smoking status (never smokers, former smokers, current light smokers [<20 cig./d], current heavy smokers [≥20 cig./d]), blood pressure, body mass index (BMI), fasting blood cholesterol and glucose, medical history (diabetes, myocardial infarction, stroke) and current medical treatment. From these variables we selected variables for inclusion in our models as explained below. Medical history was obtained with the questionnaire question: “Have you ever had or still have one of the following diseases: diabetes, myocardial infarction, stroke, hypertension?”; current medication with the questions: “Do you currently take medication for the heart or the vessels; against cholesterol or atherosclerosis?” and “If you have diabetes, what treatment do you have?”; “Medication” and “Injections” were considered as current medical treatment.

### Statistical methods

For descriptive analyses, we calculated counts, means and proportions of the variables of interest. Mortality rates by sex were age-standardized by the direct method to the WHO “European” standard age structure [15]. Cox proportional hazards regression models with adjustment for potential confounders were used to calculate hazard ratios (HRs), 95% confidence intervals (CIs) and p-values.

Survival time was defined as the time between study entry (i.e. date of examination), and either 1) date of death (from mortality records) or 2) the last potential date of death (12/31/2008 = censoring time point). Persons, who were found in the 1980 census but neither in a more recent census nor in a mortality record, were censored on 12/01/1980, and persons found in the 1990 census but neither in the 2000 census nor in a mortality record, were censored on 12/04/1990. Foreign nationals traced in the 2000 census and in an emigration record 2001–2008 were censored on the emigration date. NRP 1A participants who could neither be found in a census nor a mortality record were excluded from survival analysis. Model choice was based on Akaike's information criterion (AIC) and Bayesian information criterion (BIC). To ensure the comparability of different models via these information criteria and the comparability of their results, persons with missing values in the relevant variables were excluded before conducting the analyses.

### Statistical models

We calculated five Cox models to assess the influence of SRH on survival adjusted for an increasing number of covariates. All models were fitted to the whole data set with adjustment for sex, and also calculated separately for men and women. In model 1 we adjusted for age; in model 2 additionally for educational level and marital status; in model 3 additionally for smoking; in model 4 additionally for a combination of medical history and treatment; in model 5 additionally for fasting blood glucose and blood pressure. These two variables were preferred over cholesterol and BMI because they lead to lower AIC and BIC values. In order to achieve comparability, 49 linked participants with missing information on fasting blood glucose and/or medical history and medication were excluded in all models.

In order to test for potential confounding, sensitivity analyses were conducted by additionally adjusting for nationality, town of residence and being a sampled or spontaneous participant. In order to investigate the impact of duration of follow-up time, we also conducted separate analyses terminating the follow-up period after 2, 3, 4, … to 30 years. Due to small numbers and impending loss of power in subset analyses, we aggregated “poor” and “very poor” SRH. Analyses were performed with STATA 11 (Stata Corp, Texas, USA, 2009) except of the Kaplan-Meier-Curve that was plotted with R (R Foundation for Statistical Computing, 2.13.0).

## Results

### NRP 1A study population vs. 1980 census population

Because of the stratified sampling procedure and the lack of population weights in the original study, generalisability of the NRP 1A population had to be evaluated by a comparison with the 1980 census (Table 1). For this purpose we restricted for this table to the NRP 1A official age range and thus excluded 288 participants exceeding the upper age limit at baseline (see above, Methods, NRP 1A data).

On average, those who were sampled were younger and more frequently foreign nationals than spontaneous participants and the census population of the study towns (Table 1). They also tended to be less often females and to live in slightly smaller households. Sampled and even more spontaneous participants had less frequently a lower educational level than the census population.

### Self-rated health

As shown in Table 2, there were only few persons in the lowest SRH category. About half of the participants rated their health as “good”. Compared to the other participants, persons rating their health as “less than good” were more frequently older persons and foreign nationals and had more often an intermediate or lower educational level. Except of fewer single and more divorced and separated persons in the lower SRH categories, there were no substantial differences regarding marital status. An almost continuous increasing prevalence between “excellent” and “poor” SRH can be observed for current heavy smoking, the presence of chronic conditions and partially also for clinical risk factors. The largest differences were found for medication with more than tenfold proportions in those with “very poor” compared to those with “excellent” SRH. In regression analysis, mortality risk continuously increased between “excellent” and “very poor” SRH (Table 3, confidence intervals for Model 2–4 are given in Table S2, Supporting Information). In general, this gradient applied to men and women. However, HR increase for “good” compared to “excellent” SRH reached statistical significance in both – the basic and the fully adjusted model – only in women.

**Table 2:** Characteristics of linked NRP 1A participants by self-rated health category and sex, n = 8,008, 5 Swiss towns, 1977–79, ≥16 years at baseline.

	Men						Women					
	Excellent	Good	Fair	Poor	Very poor	I don't know	Excellent	Good	Fair	Poor	Very poor	I don't know
Participants (n)	952	1839	712	80	9	70	820	2219	1004	128	6	120
*Participants (in % of total n, by sex)*	*26.0*	*50.2*	*19.4*	*2.2*	*0.2*	*1.9*	*19.1*	*51.6*	*23.4*	*3.0*	*0.1*	*2.8*
Dropped due to missing information (n)*	7	7	6	0	0	14	3	6	3	0	0	3
Mean age (years)	39.2	42.1	45.1	50.3	48.1	39.2	39.2	43.4	45.9	49.3	48.7	44.3
Mean follow-up time (years)	26.7	25.4	22.8	21.1	18.6	20.5	27.9	26.7	25.1	24.1	20.3	25.7
Foreign nationality (%)	18.4	19.5	31.2	40.0	55.6	41.4	13.4	13.6	24.9	26.8	82.2	36.1
Mortality (all causes)												
Deaths (n)	234	602	311	42	6	23	147	609	341	54	1	36
*Deaths (in % of total n, by sex)*	*19.2*	*49.4*	*25.5*	*3.4*	*0.5*	*1.9*	*12.4*	*51.3*	*28.7*	*4.5*	*0.1*	*3.0*
Person-years (py)	25628	46744	16395	1686	167	1721	22990	59486	25318	3084	122	3161
Age standardized rate (per 100,000 py)	892	984	1449	1441	2547	2230	437	593	731	818	593	802
Socio-economic position												
Educational level												
Upper (%)	23.7	20	16.9	13.8	0	7.1	16.1	11.6	10.2	6.3	0	5.8
Intermediate (%)	46.5	47.7	46.5	32.5	22.2	55.7	52.9	45.5	39.4	26.6	16.7	31.7
Lower (%)	29.7	32.2	36.7	53.8	77.8	37.1	31	42.9	50.4	67.2	83.3	62.5
Marital status												
Married (%)	73.1	73.7	74.9	80	66.7	72.9	63.3	66.9	64.6	68	66.7	65
Widowed (%)	0.5	1.5	2.4	1.2	0	0	5.4	8.5	10.1	5.5	16.7	6.7
Divorced or separated (%)	2.7	3.9	3.8	3.8	11.1	2.9	3.5	4.9	4.8	10.9	16.7	6.7
Single (%)	23.6	20.9	19	15	22.2	24.3	27.8	19.7	20.5	15.6	0	21.7
Lifestyle												
Never smokers (%)	35.4	30.1	27	18.8	11.1	20	64.2	63.8	65.3	60.2	66.7	62.5
Former smokers (%)	18.3	18.6	20.8	18.8	0	15.7	9	8	5.8	5.5	0	5.8
Current light smokers (<20 cig./d, %)	27	26.9	23.5	33.8	55.6	25.7	21.5	20.7	19	20.3	16.7	23.3
Current heavy smokers (≥20 cig./d, %)	19.3	24.4	28.8	28.8	33.3	38.6	5.4	7.4	9.9	14.1	16.7	8.3
Medical history and medication												
Diabetes (%)	1.3	2.4	5.7	14.5	25	4.9	0.6	2.2	4	5.4	0	2
Myocardial infarction (%)	0.2	0.9	3.9	18.2	12.5	1.7	0.1	0.8	1.5	8	0	1.9
Stroke (%)	0	0.1	1.2	1.6	0	1.7	0.4	0.2	1	2.8	0	2
Currently under medication (%)	5.3	12.2	29.8	55.1	77.8	15.9	6	17.2	36.2	69.8	83.3	30.4
Clinical risk factors												
Blood pressure (mean systolic, mmHg)	129	130	131	132	127	129	122	125	126	129	122	125
Fasting blood glucose (mean, mmol/l)	5.4	5.4	5.6	5.9	4.8	5.5	5.2	5.2	5.3	5.4	5.7	5.3

*for the variables marital status, medical history and medication, blood glucose and blood pressure.

**Table 3:** Adjusted hazard ratios for all-cause mortality by self-rated health category and sex, n = 7,959, 5 Swiss towns, 1977–79, ≥16 years at baseline.

	Model 1“Basic”		Model 2“Socio-demo-graphic”	Model 3“Life-style”	Model 4“Medical history”	Model 5“Clinical”	
	HR	(95% CI)	HR	HR	HR	HR	(95% CI)
Men (n = 3,662; 1,218 deaths)							
Excellent	1		1	1	1	1	
Good	1.14	(0.98–1.32)	1.12	1.09	1.07	1.07	(0.92–1.24)
Fair	1.61	(1.36–1.91)	1.54	1.51	1.42	1.41	(1.18–1.68)
Poor	1.91	(1.38–2.66)	1.90	1.78	1.65	1.61	(1.15–2.25)
Very poor	3.32	(1.47–7.46)	3.10	2.94	2.58	2.85	(1.25–6.51)
I don't know	2.38	(1.55–3.65)	2.34	2.09	2.04	1.87	(1.21–2.88)
Covariates							
Age (per 1 additional year)*	1.09	(1.09–1.10)	1.10	1.10	1.10	1.09	(1.09–1.10)
Upper educational level			1	1	1	1	
Intermediate educational level			1.19	1.18	1.18	1.15	(0.97–1.36)
Lower educational level			1.31	1.29	1.30	1.27	(1.06–1.50)
Married			1	1	1	1	
Widowed			1.27	1.24	1.22	1.21	(0.91–1.62)
Divorced or separated			1.46	1.40	1.39	1.41	(1.04–1.90)
Single			1.48	1.52	1.51	1.47	(1.21–1.77)
Never smokers				1	1	1	
Former smokers				1.14	1.13	1.10	(0.93–1.30)
Current light smokers (<20 cig./d)				1.22	1.21	1.23	(1.05–1.44)
Current heavy smokers (≥20 cig./d)				1.75	1.76	1.76	(1.51–2.06)
No mention of medical history or medication					1	1	
Any mention of medical history or medication					1.19	1.15	(1.00–1.32)
Blood pressure (per 1 additional mmHg)*						1.01	(1.01–1.01)
Fasting blood glucose (per 1 additional mmol/l)*						1.04	(1.00–1.07)
Women (n = 4,297; 1,188 deaths)							
Excellent	1		1	1	1	1	
Good	1.31	(1.09–1.57)	1.30	1.28	1.23	1.22	(1.01–1.46)
Fair	1.62	(1.33–1.97)	1.58	1.55	1.42	1.39	(1.14–1.70)
Poor	1.89	(1.38–2.58)	1.87	1.74	1.49	1.49	(1.07–2.06)
Very poor	1.71	(0.24–12.26)	1.78	1.56	1.23	1.30	(0.18–9.35)
I don't know	1.44	(1.00–2.07)	1.43	1.42	1.32	1.26	(0.87–1.83)
Covariates							
Age (per 1 additional year)*	1.11	(1.11–1.12)	1.11	1.11	1.11	1.11	(1.10–1.11)
Upper educational level			1	1	1	1	
Intermediate educational level			1.01	1.04	1.05	1.02	(0.83–1.26)
Lower educational level			1.18	1.20	1.22	1.19	(0.97–1.45)
Married			1	1	1	1	
Widowed			0.89	0.83	0.82	0.83	(0.63–1.09)
Divorced or separated			1.14	1.12	1.10	1.08	(0.92–1.26)
Single			1.35	1.35	1.33	1.33	(1.13–1.56)
Never smokers				1	1	1	
Former smokers				1.09	1.08	1.12	(0.86–1.46)
Current light smokers (<20 cig./d)				1.15	1.17	1.18	(0.99–1.40)
Current heavy smokers (≥20 cig./d)				2.03	2.06	2.11	(1.63–2.73)
No mention of medical history or medication					1	1	
Any mention of medical history or medication					1.27	1.27	(1.12–1.44)
Blood pressure (per 1 additional mmHg)*						1.01	(1.00–1.01)
Fasting blood glucose (per 1 additional mmol/l)*						1.08	(1.04–1.13)

*continuous variable.

Model 1 (basic): age; Model 2 (socio-demographic): basic model + education, marital status; Model 3 (lifestyle): socio-demographic model + smoking status; Model 4 (medical history): lifestyle model + disease and medication status; Model 5 (clinical): medical history model + fasting blood glucose, systolic blood pressure.

95% Confidence Intervals (95% CI) of all models (including Model 2–4) are given in Table S2 (Supporting Information).

In contrast, only in men HRs for “very poor” SRH and “don't know” remained significantly different from “excellent” SRH after full adjustment and also higher than for “poor” SRH. However, the number of persons in that category was very small. The attenuating effect of increasing adjustment on estimates was similar in men and women. Only “don't know” in women lost statistical significance after full adjustment (Model 5 vs. Model 1). The HRs generally decreased from model 1 to model 5 but predominantly remained statistically significant. In general, the HRs for covariates changed less with increasing adjustment than those for SRH. Sensitivity analyses adding the variables “town”, “nationality” or “participation status” to Model 5 resulted in virtually unchanged estimates.

Also in the fully adjusted model (Model 5), age remained by far the most important risk factor for death. Covariates potentially explaining the relationship between SRH and mortality had a smaller impact, but with the expected increased HRs for heavy smokers, lower educated and never married individuals. Interestingly, when adjusted for SRH and other covariates, the association with current light smoking and being divorced/separated reached statistical significance only in men (Model 3). In men and women, blood pressure had a strong impact on mortality risk, in women also fasting blood glucose and the summary measure of medical history and medication (Model 5). A version with adjustment for age and model specific variables only (instead of cumulative adjustment) is shown in Table S1 in the Supporting Information.

**Figure 2:**
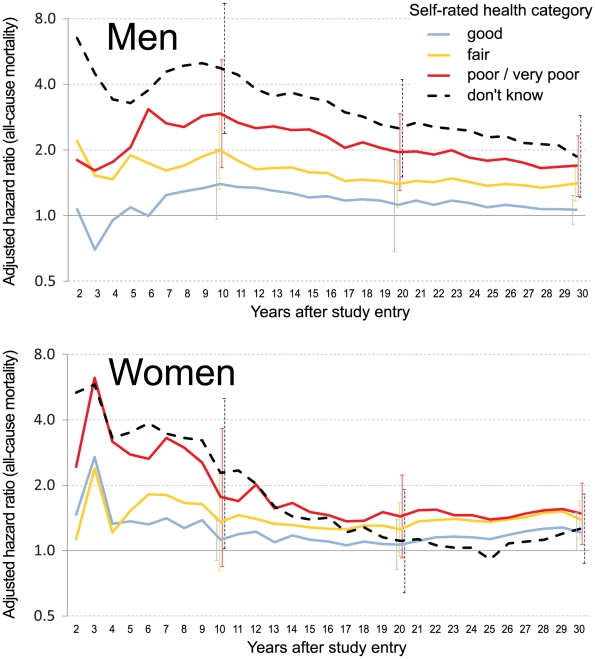
Adjusted hazard ratios for all-cause mortality, by self-rated health category, sex and increasing length of follow-up (referent: excellent SRH, n = 7,959). 95% confidence intervals are given for hazard ratios after 10 years, 20 years and maximum follow-up. Adjustment for age, marital status, educational level, smoking status, medical history, medication status, fasting blood glucose and systolic blood pressure.

Figure 2 shows the impact of follow-up time. In men, fully adjusted (corresponding to Model 5) HRs for less than “excellent” SRH reached a maximum after 9–10 years and thereafter decreased with follow-up time (Figure 2). However the ascending order of HRs didn't change and “fair”, “poor/very poor” and “don't know” significantly differed from excellent even after 30 years of follow-up. HRs in women appeared comparatively smaller and after 10 years reached statistical significance only for “don't know”. For follow-up periods between 13 and 20 years, none of the SRH categories significantly differed from “excellent”, but for periods longer than 20 years HRs for “good”, “fair” and “poor/very poor” slightly increased and eventually reached statistical significance.

**Figure 3:**
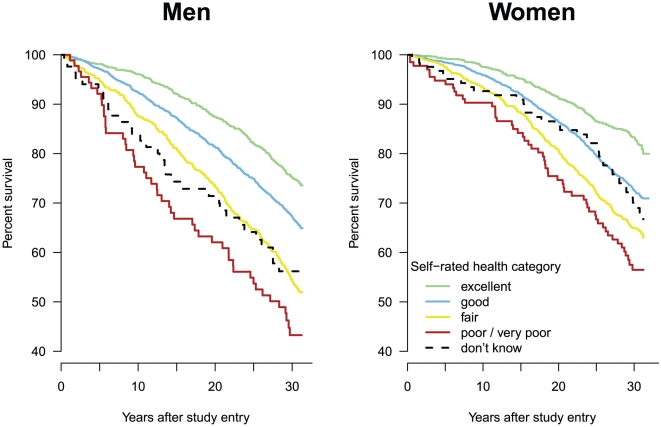
Survival of men and women by self-rated health category, Switzerland 1977–1979, followed up until 2008: Kaplan-Meier curves by sex (N = 8,008). Number of persons at risk is shown in Table S3 (Supporting Information).

As shown in Figure 3, compared to the reference group, the Kaplan Meier curves of less-than-excellent SRH decreased more strongly, suggesting rather increasing than decreasing gradients. The curve of those answering “don't know” appeared to decrease more rapidly in men than in women. Number of persons at risk is shown in Table S3 (Supporting Information).

## Discussion

Using anonymous record linkage techniques we were able to match individual data from a population based study (Switzerland, 1977–79) with national mortality records until 2008. Almost 93% of the original participants could be traced. We analysed to what extent the association between global SRH and all-cause mortality was influenced by socio-demographic, behavioural and clinical variables and examined the impact of the time passed between assessment and outcome. Even after full adjustment and considering maximum follow-up time, we found a strong and persistent “dose-dependent” relationship between SRH and all-cause mortality. To our knowledge this gradient over each of the five categories, from “very poor” to “excellent”, has not been described before. Particularly in men, answering the SRH question with “don't know” was also associated with a markedly higher mortality risk. In both sexes, this effect was strongest in the first ten years of follow-up.

In the model adjusting for age only, men with “very poor” SRH had – compared to those with “excellent” SRH – a more than threefold mortality risk. Also men and women with “poor” SRH had still a 90% mortality risk increase. This is in line with the twofold increase derived from a meta-analysis [3]. Comparisons with other studies are limited because most of them tri- or dichotomized the SRH levels and/or had a substantially shorter follow-up period [2], [6], [7], [16]. As health status may change and baseline self-ratings therefore may no longer reflect current status [1], it can be expected that SRH assessed only at baseline is a stronger predictor over shorter than over longer follow-up periods. While exclusion of the first two years of follow-up did virtually not change the results (not shown), our figures suggest a maximal gradient after about seven (women) to ten (men) years of follow-up. A maximal effect for a follow-up time of ten years was also suggested by others (differences by sex were not examined) [16].

The fact that the mortality-SRH association was gradual, independent from known clinical, behavioural and socio-demographic risk factors and persisted over more than 30 years supports rather a salutogenetic than a pathogenetic pathway: An impact of not yet diagnosed underlying disease can be imagined for a follow-up time of 5–10 years but hardly for much longer periods. Because of the persistence of the SRH-mortality association over a long period, our findings may be particularly relevant for health assessment in younger individuals. Persons rating their health as “excellent” may have an advantage over others, not primarily because of absence of disease but because of a high satisfaction with their life. In a study on subjective well-being, reporting “positive emotions” was strongly and consistently associated with lower mortality after a follow-up of 28 years [17]. Persons with such emotions may have skills to enhance health, resilience and well-being [17]. The consistent “dose-dependent” effect and particularly the statistically significant difference between those rating “excellent” and those rating “good”, support the concept of a salutogenetic rather than pathogenetic pathway.

As found by a study from Sweden [6], differences between men and women in the model adjusted for age only were marginal. However, compared to “excellent” SRH, “good” SRH was only in women significantly associated with increased mortality risk. In line with most literature [1], [2], [7], [18], males showed in the fully adjusted model a steeper gradient than women. Risk of death was also substantially increased among those with “fair” SRH. However, while in men mortality HR for “fair” SRH appeared constantly lower than for “poor” SRH, this difference tended to vanish in women with increasing follow-up time. Covariates potentially explaining the relationship between SRH and mortality had a moderate impact and only marginally attenuated with increasing adjustment. This suggests, that in contrast to physician-assessed parameters, SRH may be a dynamic (rather than cross-sectional) predictor of staying healthy and also a sustained determinant for health attitude, perception and behaviour [2].

In the adjusted models, men who answered the question about SRH with “don't know” had a HR between those with “poor” and “very poor” SRH. As shown in Table 3, adjustment for education and marital status did not attenuate the association. The fact that the “don't know”- mortality association concerned predominantly young men (not shown) suggests that “dont't know” could be a proxy for limited health perception and awareness or for risky lifestyle. However, excluding injuries from the analysis did not significantly change the association (results not shown). Most of the effect of “don't know” originated from the first 6–7 years of follow-up, suggesting the presence of severe but not yet detected diseases, i.e. in contrast to the overall pattern a pathogenetic pathway. To the best of our knowledge, in the context of global SRH, no other study mentioned this category. There were two studies on comparative SRH, addressing the higher risk of “don't know” compared to “better” SRH [7], [16]. The effect was however small [7], [16].

### Limitations

We could not adjust for health trajectories, because SRH was assessed only once (at baseline). It can be assumed that the found association with mortality may therefore be a conservative estimate, i.e. an underestimation of the real magnitude of the effect. Studies using multiple assessments of SRH and therefore allowing a dynamic evaluation show that changes in SRH are an even stronger predictor of mortality than baseline SRH [19]. Because of this, it can be expected that our figures underestimate the effect of SRH on mortality [19]. A part of the original study participants could not be linked (7.2%), emigrated (2.7%) or was lost to follow-up (3.8%, not considering the unknown figure for 2000–08). However, even on the long run, this proportion remains rather modest. Due to the design of the original study, there is some variation compared to the general population. However, sensitivity analyses did not suggest any impact of nationality, kind of recruitment or regional affiliation. In line with other health surveys, the NFP 1A participants were most likely healthier than the general population [13]. As a methodological limitation it must be mentioned that Kaplan Meier curves are useful for descriptive purposes, but do not adjust for age or other variables. Thus, the variation in decrease of the curves could be due to other factors than SRH. All the above-mentioned limitations may have some possible effect on the results. Nevertheless they are unlikely to have had any major impact on the described patterns.

### Conclusions and policy implications

Even when adjusting for age, lifestyle, socio-demographic and clinical risk factors, SRH was a strong and independent predictor of all-cause mortality in this population based cohort with a long follow-up time. In both sexes, mortality risks showed a consistent “dose-response” pattern between “excellent” and “very poor” SRH, which essentially persisted over 30 years. This substantiates the notion that this straightforward indicator really and reliably “contributes unique information that is not captured by standard clinical assessments or self reported histories” [20]. Persons rating their health with “excellent” may not primarily do so because of absence of chronic disease but rather because of distinct personal properties related to health resources.

Our findings should encourage clinicians to put more weight on the “salutogenetic” (rather than restricting to the “pathogenetic”) perspective of health [20], i.e. to not only look for the presence or absence of disease or impairment but also support their patients in managing and generating health resources and thus provide an environment that promotes a healthy life. This would be perfectly in line with the broad World Health Organization's (WHO, 1948) definition of health as “physical, mental and social well-being, not merely the absence of disease and infirmity”.

## Supporting Information

Table S1 Adjusted hazard ratios for all-cause mortality, by self-rated health category and sex, n = 7,959, Switzerland, 1977–79, ≥16 years at baseline: separate and not cumulative adjustment. Model 1 (basic): age; Model 2 (socio-demographic): age + education, marital status; Model 3 (lifestyle): age + smoking status; Model 4 (medical history): age + disease and medication status; Model 5 (clinical): age + fasting blood glucose, systolic blood pressure.
(DOC)

Table S2 Adjusted hazard ratios for all-cause mortality, by self-rated health category and sex, n = 7,959, 5 Swiss towns, 1977–79, ≥16 years at baseline. *continuous variable. Model 1 (basic): age; Model 2 (socio-demographic): basic model + education, marital status; Model 3 (lifestyle): socio-demographic model + smoking status; Model 4 (medical history): lifestyle model + disease and medication status; Model 5 (clinical): medical history model + fasting blood glucose, systolic blood pressure.
(DOC)

Table S3 Number of persons at risk at study entry and 10, 20 and 30 years thereafter, by self-rated health category and sex, n = 8,008, 5 Swiss towns, 1977–79, ≥16 years at baseline.
(DOC)
