# Supplementary material for: Health Risk or Resource? Gradual and Independent Association between Self-Rated Health and Mortality Persists Over 30 Years
Source: PLoS One. 2012 Feb 9;7(2):e30795. doi: 10.1371/journal.pone.0030795 (PMC3276505; doi:10.1371/journal.pone.0030795)
Supplement: Table S1 — Adjusted hazard ratios for all-cause mortality, by self-rated health category and sex, n = 7,959, Switzerland, 1977–79, ≥16 years at baseline: separate and not cumulative adjustment. Model 1 (basic): age; Model 2 (socio-demographic): age + education, marital status; Model 3 (lifestyle): age + smoking status; Model 4 (medical history): age + disease and medication status; Model 5 (clinical): age + fasting blood glucose, systolic blood pressure. (DOC) [file pone.0030795.s001.doc]

**Supporting Information**

**Table S1**. Adjusted hazard ratios for all-cause mortality, by self-rated health category and sex, n=7,959, Switzerland, 1977-79, ≥ 16 years at baseline: separate and not cumulative adjustment

|  |  |  |  |  |  |  |  |  |  |  |  |  |  |  |
| --- | --- | --- | --- | --- | --- | --- | --- | --- | --- | --- | --- | --- | --- | --- |
|  | Model 1 "Basic" | |  | Model 2 "Socio-demographic" | |  | Model 3 "Lifestyle" | |  | Model 4 "Medical history" | |  | Model 5 "Clinical" | |
|  | HR | *P* |  | HR | *P* |  | HR | *P* |  | HR | P |  | HR | *P* |
| Men |  |  |  |  |  |  |  |  |  |  |  |  |  |  |
| Excellent | 1 |  |  | 1 |  |  | 1 |  |  | 1 |  |  | 1 |  |
| Good | 1.14 | *0.09* |  | 1.12 | *0.137* |  | 1.11 | *0.16* |  | 1.12 | *0.135* |  | 1.13 | *0.116* |
| Fair | 1.61 | *<0.001* |  | 1.54 | *<0.001* |  | 1.59 | *<0.001* |  | 1.52 | *<0.001* |  | 1.56 | *<0.001* |
| Poor | 1.91 | *<0.001* |  | 1.90 | *<0.001* |  | 1.83 | *<0.001* |  | 1.77 | *0.001* |  | 1.84 | *<0.001* |
| Very poor | 3.32 | *0.004* |  | 3.10 | *0.006* |  | 3.13 | *0.006* |  | 2.94 | *0.01* |  | 3.64 | *0.002* |
| Don’t know | 2.38 | *<0.001* |  | 2.34 | *<0.001* |  | 2.14 | *0.001* |  | 2.31 | *<0.001* |  | 2.16 | *<0.001* |
| Women |  |  |  |  |  |  |  |  |  |  |  |  |  |  |
| Excellent | 1 |  |  | 1 |  |  | 1 |  |  | 1 |  |  | 1 |  |
| Good | 1.31 | *0.003* |  | 1.30 | *0.004* |  | 1.28 | *0.007* |  | 1.26 | *0.012* |  | 1.29 | *0.005* |
| Fair | 1.62 | *<0.001* |  | 1.58 | *<0.001* |  | 1.59 | *<0.001* |  | 1.49 | *<0.001* |  | 1.58 | *<0.001* |
| Poor | 1.89 | *<0.001* |  | 1.87 | *<0.001* |  | 1.77 | *<0.001* |  | 1.63 | *0.003* |  | 1.87 | *<0.001* |
| Very poor | 1.71 | *0.591* |  | 1.78 | *0.566* |  | 1.47 | *0.704* |  | 1.38 | *0.749* |  | 1.82 | *0.550* |
| Don’t know | 1.44 | *0.052* |  | 1.43 | *0.057* |  | 1.43 | *0.056* |  | 1.35 | *0.112* |  | 1.38 | *0.084* |

Model 1 (basic): age; Model 2 (socio-demographic): age + education, marital status; Model 3 (lifestyle): age + smoking status; Model 4 (medical history): age + disease and medication status; Model 5 (clinical): age + fasting blood glucose, systolic blood pressure
