# Supplementary material for: Health Risk or Resource? Gradual and Independent Association between Self-Rated Health and Mortality Persists Over 30 Years
Source: PLoS One. 2012 Feb 9;7(2):e30795. doi: 10.1371/journal.pone.0030795 (PMC3276505; doi:10.1371/journal.pone.0030795)
Supplement: Table S2 — Adjusted hazard ratios for all-cause mortality, by self-rated health category and sex, n = 7,959, 5 Swiss towns, 1977–79, ≥16 years at baseline. *continuous variable. Model 1 (basic): age; Model 2 (socio-demographic): basic model + education, marital status; Model 3 (lifestyle): socio-demographic model + smoking status; Model 4 (medical history): lifestyle model + disease and medication status; Model 5 (clinical): medical history model + fasting blood glucose, systolic blood pressure. (DOC) [file pone.0030795.s002.doc]

**Supporting Information**

**Table S2.** Adjusted hazard ratios for all-cause mortality, by self-rated health category and sex, n=7,959, 5 Swiss towns, 1977-79, ≥ 16 years at baseline

|  |  |  |  |  |  |  |  |  |  |  |  |  |  |  |
| --- | --- | --- | --- | --- | --- | --- | --- | --- | --- | --- | --- | --- | --- | --- |
|  | Model 1 "Basic" | |  | Model 2 "Socio-demographic" | |  | Model 3 "Lifestyle" | |  | Model 4 "Medical history" | |  | Model 5 "Clinical" | |
|  | HR | (95% CI) |  | HR | (95% CI) |  | HR | (95% CI) |  | HR | (95% CI) |  | HR | (95% CI) |
| Men (n=3,662; 1,218 deaths) |  |  |  |  |  |  |  |  |  |  |  |  |  |  |
| Excellent | 1 |  |  | 1 |  |  | 1 |  |  | 1 |  |  | 1 |  |
| Good | 1.14 | (0.98-1.32) |  | 1.12 | (0.96-1.30) |  | 1.09 | (0.94-1.27) |  | 1.07 | (0.92-1.25) |  | 1.07 | (0.92-1.24) |
| Fair | 1.61 | (1.36-1.91) |  | 1.54 | (1.30-1.83) |  | 1.51 | (1.27-1.79) |  | 1.42 | (1.19-1.70) |  | 1.41 | (1.18-1.68) |
| Poor | 1.91 | (1.38-2.66) |  | 1.90 | (1.36-2.64) |  | 1.79 | (1.28-2.49) |  | 1.65 | (1.18-2.31) |  | 1.61 | (1.15-2.25) |
| Very poor | 3.31 | (1.47-7.46) |  | 3.10 | (1.37-7.01) |  | 2.94 | (1.30-6.66) |  | 2.58 | (1.13-5.88) |  | 2.85 | (1.25-6.51) |
| I don't know | 2.38 | (1.55-3.65) |  | 2.34 | (1.52-3.59) |  | 2.09 | (1.36-3.22) |  | 2.04 | (1.32-3.14) |  | 1.87 | (1.21-2.88) |
| Covariates |  |  |  |  |  |  |  |  |  |  |  |  |  |  |
| Age (per 1 additional year)* | 1.09 | (1.09-1.10) |  | 1.10 | (1.09-1.10) |  | 1.10 | (1.09-1.10) |  | 1.10 | (1.09-1.10) |  | 1.09 | (1.09-1.10) |
| Upper educational level |  |  |  | 1 |  |  | 1 |  |  | 1 |  |  | 1 |  |
| Intermediate educational level |  |  |  | 1.19 | (1.01-1.41) |  | 1.18 | (0.99-1.39) |  | 1.18 | (1.00-1.39) |  | 1.15 | (0.97-1.36) |
| Lower educational level |  |  |  | 1.31 | (1.10-1.56) |  | 1.29 | (1.09-1.53) |  | 1.29 | (1.09-1.54) |  | 1.27 | (1.06-1.50) |
| Married |  |  |  | 1 |  |  | 1 |  |  | 1 |  |  | 1 |  |
| Widowed |  |  |  | 1.27 | (0.96-1.69) |  | 1.24 | (0.94-1.66) |  | 1.23 | (0.92-1.63) |  | 1.21 | (0.91-1.62) |
| Divorced or separated |  |  |  | 1.46 | (1.08-1.98) |  | 1.40 | (1.04-1.90) |  | 1.39 | (1.03-1.89) |  | 1.41 | (1.04-1.90) |
| Single |  |  |  | 1.48 | (1.23-1.79) |  | 1.52 | (1.26-1.84) |  | 1.51 | (1.25-1.83) |  | 1.47 | (1.21-1.77) |
| Never smokers |  |  |  |  |  |  | 1 |  |  | 1 |  |  | 1 |  |
| Former smokers |  |  |  |  |  |  | 1.14 | (0.97-1.35) |  | 1.13 | (0.95-1.33) |  | 1.10 | (0.93-1.30) |
| Current light smokers (<20 cig./d) |  |  |  |  |  |  | 1.22 | (1.04-1.43) |  | 1.21 | (1.04-1.42) |  | 1.23 | (1.05-1.44) |
| Current heavy smokers (?20 cig./d) |  |  |  |  |  |  | 1.75 | (1.50-2.04) |  | 1.76 | (1.50-2.05) |  | 1.76 | (1.51-2.06) |
| No mention of medical history or medication |  |  |  |  |  |  |  |  |  | 1 |  |  | 1 |  |
| Any mention of medical history or medication |  |  |  |  |  |  |  |  |  | 1.19 | (1.04-1.37) |  | 1.15 | (1.00-1.32) |
| Blood pressure (per 1 additional mmHg)* |  |  |  |  |  |  |  |  |  |  |  |  | 1.01 | (1.01-1.01) |
| Fasting blood glucose (per 1 additional mmol/l)* |  |  |  |  |  |  |  |  |  |  |  |  | 1.04 | (1.00-1.07) |

|  |  |  |  |  |  |  |  |  |  |  |  |  |  |  |
| --- | --- | --- | --- | --- | --- | --- | --- | --- | --- | --- | --- | --- | --- | --- |
|  | Model 1 "Basic" | |  | Model 2 "Socio-demographic" | |  | Model 3 "Lifestyle" | |  | Model 4 "Medical history" | |  | Model 5 "Clinical" | |
|  | HR | (95% CI) |  | HR | (95% CI) |  | HR | (95% CI) |  | HR | (95% CI) |  | HR | (95% CI) |
| Women (n=4,297; 1,188 deaths) |  |  |  |  |  |  |  |  |  |  |  |  |  |  |
| Excellent | 1 |  |  | 1 |  |  | 1 |  |  | 1 |  |  | 1 |  |
| Good | 1.31 | (1.09-1.57) |  | 1.30 | (1.09-1.56) |  | 1.28 | (1.07-1.53) |  | 1.23 | (1.02-1.48) |  | 1.22 | (1.01-1.46) |
| Fair | 1.62 | (1.33-1.97) |  | 1.58 | (1.30-1.92) |  | 1.55 | (1.28-1.88) |  | 1.42 | (1.16-1.73) |  | 1.39 | (1.14-1.70) |
| Poor | 1.89 | (1.38-2.58) |  | 1.87 | (1.36-2.56) |  | 1.74 | (1.27-2.39) |  | 1.49 | (1.08-2.07) |  | 1.49 | (1.07-2.06) |
| Very poor | 1.71 | (0.24-12.26) |  | 1.78 | (0.25-12.75) |  | 1.56 | (0.22-11.18) |  | 1.23 | (0.17-8.89) |  | 1.30 | (0.18-9.35) |
| I don't know | 1.44 | (1.00-2.07) |  | 1.43 | (0.99-2.06) |  | 1.42 | (0.98-2.04) |  | 1.32 | (0.91-1.90) |  | 1.26 | (0.87-1.83) |
| Covariates |  |  |  |  |  |  |  |  |  |  |  |  |  |  |
| Age (per 1 additional year)* | 1.11 | (1.11-1.12) |  | 1.11 | (1.10-1.12) |  | 1.11 | (1.11-1.12) |  | 1.11 | (1.11-1.12) |  | 1.11 | (1.10-1.11) |
| Upper educational level |  |  |  | 1 |  |  | 1 |  |  | 1 |  |  | 1 |  |
| Intermediate educational level |  |  |  | 1.01 | (0.82-1.25) |  | 1.04 | (0.84-1.28) |  | 1.05 | (0.85-1.30) |  | 1.02 | (0.83-1.26) |
| Lower educational level |  |  |  | 1.18 | (0.96-1.44) |  | 1.20 | (0.98-1.47) |  | 1.22 | (1.00-1.49) |  | 1.19 | (0.97-1.45) |
| Married |  |  |  | 1 |  |  | 1 |  |  | 1 |  |  | 1 |  |
| Widowed |  |  |  | 0.89 | (0.68-1.16) |  | 0.83 | (0.63-1.09) |  | 0.82 | (0.63-1.08) |  | 0.83 | (0.63-1.09) |
| Divorced or separated |  |  |  | 1.14 | (0.98-1.34) |  | 1.12 | (0.96-1.31) |  | 1.10 | (0.95-1.29) |  | 1.08 | (0.92-1.26) |
| Single |  |  |  | 1.35 | (1.15-1.59) |  | 1.35 | (1.15-1.59) |  | 1.34 | (1.14-1.57) |  | 1.33 | (1.13-1.56) |
| Never smokers |  |  |  |  |  |  | 1 |  |  | 1 |  |  | 1 |  |
| Former smokers |  |  |  |  |  |  | 1.09 | (0.84-1.42) |  | 1.08 | (0.83-1.40) |  | 1.12 | (0.86-1.46) |
| Current light smokers (<20 cig./d) |  |  |  |  |  |  | 1.16 | (0.97-1.38) |  | 1.17 | (0.98-1.39) |  | 1.18 | (0.99-1.40) |
| Current heavy smokers (?20 cig./d) |  |  |  |  |  |  | 2.03 | (1.57-2.63) |  | 2.06 | (1.60-2.67) |  | 2.11 | (1.63-2.73) |
| No mention of medical history or medication |  |  |  |  |  |  |  |  |  | 1 |  |  | 1 |  |
| Any mention of medical history or medication |  |  |  |  |  |  |  |  |  | 1.27 | (1.12-1.44) |  | 1.27 | (1.12-1.44) |
| Blood pressure (per 1 additional mmHg)* |  |  |  |  |  |  |  |  |  |  |  |  | 1.01 | (1.00-1.01) |
| Fasting blood glucose (per 1 additional mmol/l)* |  |  |  |  |  |  |  |  |  |  |  |  | 1.08 | (1.04-1.13) |

*continuous variable

Model 1 (basic): age; Model 2 (socio-demographic): basic model + education, marital status; Model 3 (lifestyle): socio-demographic model + smoking status; Model 4 (medical history): lifestyle model + disease and medication status; Model 5 (clinical): medical history model + fasting blood glucose, systolic blood pressure
