# Supplementary material for: Health Risk or Resource? Gradual and Independent Association between Self-Rated Health and Mortality Persists Over 30 Years
Source: PLoS One. 2012 Feb 9;7(2):e30795. doi: 10.1371/journal.pone.0030795 (PMC3276505; doi:10.1371/journal.pone.0030795)
Supplement: Table S3 — Number of persons at risk at study entry and 10, 20 and 30 years thereafter, by self-rated health category and sex, n = 8,008, 5 Swiss towns, 1977–79, ≥16 years at baseline. (DOC) [file pone.0030795.s003.doc]

**Supporting Information**

**Table S3.** Number of persons at risk at study entry and 10, 20 and 30 years thereafter, by self-rated health category and sex, n=8,008, 5 Swiss towns, 1977-79, ≥ 16 years at baseline

|  |  |  |  |  |  |  |  |  |  |
| --- | --- | --- | --- | --- | --- | --- | --- | --- | --- |
|  | Years after study entry, men | | | |  | Years after study entry, women | | | |
| Self-rated health | 0 | 10 | 20 | 30 |  | 0 | 10 | 20 | 30 |
| Excellent | 959 | 908 | 781 | 648 |  | 823 | 795 | 710 | 622 |
| Good | 1846 | 1687 | 1404 | 1107 |  | 2225 | 2113 | 1821 | 1458 |
| Fair | 718 | 618 | 470 | 298 |  | 1007 | 930 | 756 | 537 |
| Poor & very poor | 89 | 68 | 52 | 29 |  | 134 | 121 | 94 | 55 |
| Don't know | 84 | 66 | 49 | 35 |  | 123 | 113 | 97 | 66 |
